# A Genome-Wide Analysis of RNA Pseudoknots That Stimulate Efficient −1 Ribosomal Frameshifting or Readthrough in Animal Viruses

**DOI:** 10.1155/2013/984028

**Published:** 2013-11-04

**Authors:** Xiaolan Huang, Qiang Cheng, Zhihua Du

**Affiliations:** ^1^Department of Computer Science, Southern Illinois University Carbondale, IL 62901, USA; ^2^Department of Chemistry and Biochemistry, Southern Illinois University Carbondale, IL 62901, USA

## Abstract

Programmed −1 ribosomal frameshifting (PRF) and stop codon readthrough are two translational recoding mechanisms utilized by some RNA viruses to express their structural and enzymatic proteins at a defined ratio. Efficient recoding usually requires an RNA pseudoknot located several nucleotides downstream from the recoding site. To assess the strategic importance of the recoding pseudoknots, we have carried out a large scale genome-wide analysis in which we used an in-house developed program to detect all possible H-type pseudoknots within the genomic mRNAs of 81 animal viruses. Pseudoknots are detected downstream from ~85% of the recoding sites, including many previously unknown pseudoknots. ~78% of the recoding pseudoknots are the most stable pseudoknot within the viral genomes. However, they are not as strong as some designed pseudoknots that exhibit roadblocking effect on the translating ribosome. Strong roadblocking pseudoknots are not detected within the viral genomes. These results indicate that the decoding pseudoknots have evolved to possess optimal stability for efficient recoding. We also found that the sequence at the *gag-pol* frameshift junction of HIV1 harbors potential elaborated pseudoknots encompassing the frameshift site. A novel mechanism is proposed for possible involvement of the elaborated pseudoknots in the HIV1 PRF event.

## 1. Introduction

During the translation process, ribosomes are capable of performing some nonstandard decoding events which provided that appropriate signals are present in the mRNA being translated. These unusual events are referred to as “recoding” [1, 2]. Two of the major recoding mechanisms are programmed −1 ribosomal frameshifting (PRF) and stop codon readthrough. These mechanisms are utilized by retroviruses and some other RNA viruses to express their structural and enzymatic proteins at a defined ratio [1, 3–5]. Both −1 frameshifting and stop codon readthrough are site specific and occur at a defined frequency much higher than the background error rates of maintaining the reading frames.

**Figure 1:**
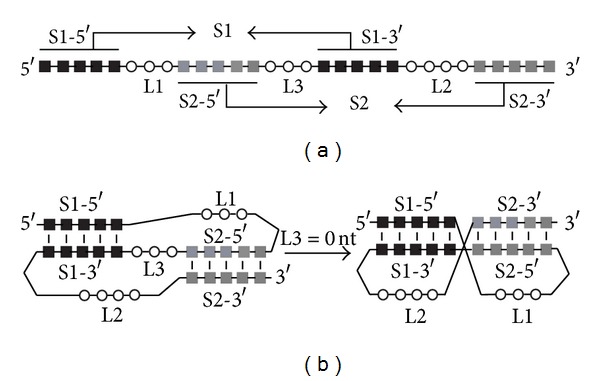
Schematic diagrams of the sequence elements for forming an H-type RNA pseudoknot. Abbreviations used are: S1, stem1; S2, stem2; S1-5′ and S1-3′, the 5′ and 3′ strands of stem1; S2-5′ and S2-3′, the 5′ and 3′ strands of stem2; L1, loop1; L2, loop2; L3, loop3. (a): Linear sequential arrangement of the pseudoknot-forming sequence elements. Residues involved in the formation of S1 and S2 are represented as black and gray squares, respectively. Residues in the single-stranded loop region are represented as unfilled circles. (b): Schematic representations of folded pseudoknots. Left: with a nonzero L3 sequence; right: with the absence of L3, S1 and S2 can stack coaxially to form a quasicontinuous double helix. L1 and L2 locate on the same side of the double helix, with L1 crossing the major groove of S2 and L2 crossing the minor groove of S1.

The discovery of the −1 PRF mechanism was made by Atkins and coworkers [6], and the utilization of this recoding mechanism by viruses was described as a strategy by which Rous sarcoma virus (RSV) expresses its *gag-pol* polyprotein from the overlapping *gag* and *pol* open reading frames from a single translation initiation codon of the 5′*gag* reading frame [7]. In −1 frameshifting, only a defined percentage of the translating ribosomes shifts to the −1 reading frame and translates the downstream gene. This percentage is referred to as the frameshifting efficiency, which dictates the molar ratio of viral structural and enzymatic proteins, encoded by the *gag* and *pol* gene, respectively. For efficient −1 frameshifting to happen, two *cis-*acting elements programmed in the overlapping region of the mRNA are often required to signal the translating ribosomes to shift backward by one nucleotide. The first element is a heptanucleotide stretch termed “slippery sequence” with a typical composition of X XXY YYZ (XXX and YYY: a stretch of three identical nucleotides; the triplets indicate the 0 reading frame). Although the slippery sequence is the site of action where the ribosomes actually shift to the −1 frame, this element alone is not sufficient to cause efficient shifting. A secondary signal called “stimulator” is usually required in the form of an RNA structure downstream from the frameshift site, separated by a spacer region (typically 6–9 nucleotides in length). In some cases, the stimulator is a conventional stem-loop structure, but most often it is a pseudoknot, which is a structural motif of RNA formed when a stretch of nucleotides within a loop region in a secondary structure basepairs with residues outside that loop [8–10] (Figure 1). The indispensable role of a downstream pseudoknot in efficient −1 frameshifting has been established in a large number of RNA viruses including members from the retroviridae family, coronaviridae family (such as SARS CoV), totiviridae family, and Luteoviridae family [5, 11–18]. Although a 3′ RNA structure is utilized as a frameshift stimulator in most cases, absence of such a structure has also been reported in efficient frameshifting such as in the case of Semliki Forest virus [19].

 The involvement of RNA pseudoknots in stop codon readthrough has also been established. In the *gag-pol* junction region of Moloney murine leukemia virus (Mo-MuLV), a pseudoknot located several nucleotides 3′ to the UAG termination codon of the *gag *gene was found to be required [20, 21]. Strong similarities between the sequences in the *gag-pol* region of MuLV and the other viruses of the readthrough retrovirus group imply that the other readthrough retroviruses may use a similar pseudoknot structure to stimulate the stop codon readthrough as well [22, 23]. 

 The vast majority of the established frameshift- or readthrough-stimulating pseudoknots belong to the so-called H (hairpin)-type pseudoknots, in which a stretch of nucleotides within a hairpin loop basepairs with a complementary region outside of the hairpin (see Figure 1 for the secondary structure and terminology of an H-type pseudoknot). All H-type pseudoknots contain two helical stems, S1 and S2, and two nonequivalent loops, L1 and L2. Some H-type pseudoknots also contain a third loop, L3. If L3 is absent, S1 and S2 can form a quasicontinuous double helix, with loops L1 and L2 crossing the major groove and minor groove of stem S2 and stem S1, respectively (Figure 1). 

 The structures of many −1 frameshift stimulating pseudoknots have been determined by NMR or X-ray crystallography, including those at the *gag-pro *junctions from the mouse mammary tumor virus (MMTV) (24–26) and the simian retrovirus-1 (SRV-1) [24, 25], the P1-P2 junctions from several plant Luteoviruses: the beet western yellows virus (BWYV) [26–28], the pea enation mosaic virus (PEMV-1) [29], the potato leaf roll virus (PLRV) [30], and the sugar cane yellow leaf virus (ScYLV) [31]. In the MMTV pseudoknot, L3 consists of an unpaired adenosine that is intercalated between the two stems S1 and S2 thereby inducing a bent conformation of the pseudoknot. In the SRV-1 pseudoknot, L3 is absent, and the two stems S1 and S2 stack coaxially to form a quasi-continuous helix. The luteoviral pseudoknots are small and compact (with only 4-5 basepairs in S1 and 3 basepairs in S2). Extensive S1-L2 and S2-L1 interactions are present in all of the luteoviral pseudoknots. Overall, the available structures do not share common structural feature(s) other than the fact that they all adopt the general pseudoknotted topology. These results indicate that the frameshift stimulating ability of a pseudoknot is not dependent on its specific or unique structural feature(s). Several lines of evidence suggest that the thermodynamic stability and mechanical strength of a frameshift stimulating pseudoknot to resist unwinding by the helicase activity of the ribosome may correlate more strongly with frameshift stimulation.

 It is known that some mRNA structures, especially pseudoknots, can cause the translating ribosome to pause upstream to such structures [32–34]. In a low resolution (~16 Å) structure, obtained by cryoelectron microscopy, of mammalian 80S ribosome in complex with the infectious bronchitis virus (IBV) pp1a/pp1b pseudoknot frameshift signal, as well as eEF2 occupying the A-site and a tRNA occupying the P-site [35], it is found that the paused pseudoknot is stuck at the entrance of the mRNA tunnel of the ribosome, and the D-helix of the P-site tRNA bends heavily toward the 3′ direction (compared to a structure with nonframeshift stimulating stem-loop RNA). The opposing forces placed by translocation and the wedged pseudoknot may create local tension on the mRNA, which can be relaxed by frameshifting. The superior ability of pseudoknots to pause ribosomes is presumably due to the unique pseudoknotted topology. The presence of stem2 greatly limits the rotational freedom of stem1, making the pseudoknots harder to unwind by the translating ribosomes than simple stem–loop structures with comparable thermodynamic stability [36]. 

 Single molecule studies using optical tweezers to pull pseudoknots apart [37–39] showed that the mechanical force required to unfold pseudoknots is much larger than the Gibbs free-energy difference (Δ*G*) between folded and unfolded pseudoknots. The requirement for extra energy input may explain why the pseudoknot is more resistant to unfolding by optical tweezers (presumably by ribosomes as well). The mechanical strength of pseudoknots was further correlated with the frameshift stimulating ability of the pseudoknots [37, 38]. By extrapolating the data, it was proposed that pseudoknots with certain mechanical strength would be able to stimulate −1 frameshifting of 100% efficiency, and pseudoknots with even higher mechanical strength would stall the ribosomes completely like a roadblock (with or without frameshifting), leading to translation termination [38]. 

 A more recent study confirmed the ribosomal roadblocking effect of strong pseudoknots [40]. By investigating a number of designed pseudoknots with varied numbers of basepairs in the stems, it was shown that the strongest pseudoknots (as predicted) only induced limited frameshifting, as judged by the amount of full-length frameshifted products. However, analysis of the pulse labeled proteins revealed that a significant fraction of the ribosomes did shift frames but failed to pass the pseudoknot structures to continue translation; the strength of the pseudoknots correlated not only with the fraction of frameshifted ribosomes but also the roadblocking effect. Based on these observations, it was proposed that the optimal frameshifting efficiency would be produced when a balance of the two effects is achieved. According to this hypothesis, the naturally occurring frameshift stimulating pseudoknots should have optimal mechanical strength to cause the right amount of translating ribosomes to shift frames but should not be too strong in order to ensure that the ribosomes are not stalled permanently. It is also implied that strong pseudoknots that exhibit a roadblocking effect should not be present in the coding regions of mRNAs.

 Given these recent progresses on the mechanisms of frameshifting, it would be interesting to assess the uniqueness of the frameshift stimulating pseudoknots in the viral genomic RNAs. How many other potential pseudoknots are present in the viral mRNAs? How do the other pseudoknots compare to the frameshift stimulating pseudoknots in terms of thermodynamic stability and mechanical strength? Are there strong roadblocking pseudoknots in the viral genomic mRNAs? To address these questions, we have developed a computer program capable of identifying potential H-type pseudoknots in any given mRNA sequence and ranking the identified pseudoknots according to the relative strength of their helical stems. Using the program, we have analyzed the full-length genomic mRNAs of 81 animal viruses that are known or expected to use −1 frameshifting or readthrough as a decoding mechanism for protein expression. 

## 2. Materials and Methods

A computer program has been developed to identify all putative H-type pseudoknots within any given RNA sequence (Bioinformation, in press).  Figure 1(a) shows a linear presentation of the sequence elements of a typical H-type pseudoknot, which requires that both helical stems (S1 and S2) can form simultaneously. If a given RNA sequence contains two pairs of complementary stretches (S1-5′ complementary to S1-3′ and S2-5′ complementary to S2-3′) G-U is considered a legitimate basepair separated by two or three connecting unpaired regions (L1, L2, and optionally L3) with a sequential arrangement as shown in Figure 1(a), then an H-type pseudoknot can potentially form within this sequence. The computer program tests all possible combinations of stem and loop lengths within certain ranges to see whether the pseudoknot-forming criteria can be met. The ranges for the lengths of the stems and loops can be set by the user. The default ranges are as follows: S1 has 5 to 20 base pairs; S2 has 5 to 20 base pairs; L1 has 1 to 10 nucleotides; L2 has 3 to 50 nucleotides, and L3 has 0 to 10 nucleotides. 

 In order to compare the relative thermodynamic stability and mechanical strength of the identified pseudoknots within a given mRNA sequence, we implemented free energy (Δ*G*_37_°) calculation for the two helical stems S1 and S2. In calculating the free energy, the Turner's nearest-neighbor parameters are used [41]. If L3 = 0, the two stems are taken as a continuous helical stem for the calculation but only half of the value is given to the S1-S2 stack to account for the quasi-continuous nature of the stacked stems. If an L3 is present, the free energy is calculated as a sum of the energies of the two individual stems. Although this simplified free energy calculation should only be viewed as semi quantitative, it provides a reasonable estimation of the relative stability of the detected pseudoknots, which are ranked according to the calculated free energies. The calculated free energy value is also used as a criterion to discard those pseudoknots with less stable stems. By default, only those pseudoknots with a free energy value lower than −18 kcal/mol are kept for further analysis.

 The output file of the program contains information about whether pseudoknots are found and how many are found; the detected pseudoknots are then listed in the order of calculated free energy of the stems. For each of the detected pseudoknots, the following information is given: lengths of S1, S2, L1, L2, and L3; free energy value of the stems; size and location of the pseudoknot. A schematic diagram is then drawn showing the actual pseudoknot forming sequence and base pairing of the two stems; a sequence of 20 nucleotides immediately 5′ - to the pseudoknot is also shown because frameshift or readthrough pseudoknots usually appear several nucleotides downstream of the frameshift or readthrough sites (see Supplementary File 1, for an example output file in Supplementary Materials available online at http://dx.doi.org/10.1155/2013/984028). 

### 2.1. Viruses and Their Sequence Sources

 Most of the viruses and their sequence sources investigated in this study are taken from Recode V2.0: Database of translational recoding events [42]. The names, abbreviations and sequence IDs for the viruses are listed below. *Arteriviridae family*: Equine arteritis virus (EAV, NC_002532), Lelystad virus (LV, M96262), Lactate dehydrogenase-elevating virus (LDV, NC_002534), Porcine reproductive and respiratory syndrome virus (PRRSV, AF046869), and Simian hemorrhagic fever virus (SHFV, NC_003092). *Coronaviridae family*: Transmissible gastroenteritis virus (TGEV, NC_002306), Bovine coronavirus (BCV, NC_003045), Human coronavirus 229E (HCoV-229E, NC_002645), Human coronavirus OC43 (HCoV-OC43, NC_005147), Porcine epidemic diarrhea virus (PEDV, NC_003436), Human coronavirus HKU1 (HCoV-HKU1, AY597011), Murine hepatitis coronavirus (MHV, AF029248), SARS coronavirus (SARS-CoV, NC_004718), avian infectious bronchitis coronavirus (IBV, M95169), Bovine torovirus (Breda virus) (BRV, NC_007447), and Equine torovirus (Berne Virus) (BEV, X52374). *Astroviridae family*: Chicken astrovirus (CAstV, NC_003790), Turkey astrovirus (TAstV, NC_002470), Human astrovirus (HAstV, L13745), Mink astrovirus (MAstV, NC_004579), and Ovine astrovirus (OAstV, NC_002469). *Flaviviridae family*: Japanese encephalitis virus (JEV, NC_001437), Murray Valley encephalitis virus-Alfuy (ALFV, AY898809), Murray Valley encephalitis virus (MVEV, NC_000943), Usutu virus (USUV, NC_006551), West Nile virus-Kunjin (WNVKUN, AY274504), West Nile virus H442 (WNV-H442, EF429200), West Nile virus (WNV, NC_009942). *Retroviridae family*: avian leukosis virus HPRS-103 (ALV, NC_001408); Rous sarcoma virus (RSV, AF033808); jaagsiekte sheep retrovirus (JSRV, NC_001494); Mason-Pfizer monkey virus (MPMV, NC_001550); simian retroviruses type-1 (SRV-1, M11841) and type-2 (SRV-2, AF126467); mouse mammary tumor virus (MMTV, M15122); squirrel monkey retrovirus (SMRV-H, NC_001514); human endogenous retrovirus K10 (HERV-K10, M14123); three intracisternal A particle (IAP) genetic elements from Chinese hamster (CHIAP34, M73970), Syrian hamster (IAP-H18, M10134), and mouse (m-IAP, M17551); bovine immunodeficiency virus (BIV, NC_001413); human T-cell leukemia virus type-I (HTLV-I, AF033817) and type-II (HTLV-II, M10060); simian T-cell leukemia virus type-I (STLV-I, NC_000858); Walleye dermal sarcoma virus AF033822; baboon endogenous virus (BaEV, D10032); Feline leukemia virus (FeLV, AF052723); gibbon ape leukemia virus (GaLV, M26927); Moloney murine sarcoma virus (MSV, AF033813); Moloney murine leukemia virus (Mo-MuLV, AF033811); Friend murine leukemia virus (F-MuLV, NC_001362); Akv murine leukemia virus (Akv-MuLV, J01998); bovine leukemia virus (BLV, NC_001414); Jembrana disease virus (JDV, U21603); Ovine lentivirus (OvLV, NC_001511); South African Ovine Maedi Visna virus (SA-OMVV, AF033815); Caprine Arthritis Encephalitis virus (CAEV, NC_001463); feline immunodeficiency virus (FIV, M25381); equine infectious anaemia virus (EIAV, AF033820); puma lentivirus (PLV-14, PLU03982); human immunodifeciency virus type 1 strain (HXB2, K03455); human immunodeficiency virus type 2 (HIV-2, NC_001722); simian immunodeficiency virus (SIV, M66437, NC_004455). *Togaviridae family*: Aura virus (AURAV, NC_003900); Barmah Forest virus (BFV, NC_001786); Chikungunya virus (CHIKV, NC_004162); Eastern equine encephalitis virus (EEEV, NC_003899), Fort Morgan virus (FMV, AF339474); Getah virus (GETV, NC_006558); Highlands J virus (HJV, NC_012561); Mayaro virus (MAYV, NC_003417); Middelburg virus (MIDV, EF536323); Ndumu virus (NDUV, AF339487); O'nyong-nyong virus (ONNV, NC_001512); Ross River virus (RRV, NC_001544); Salmon pancreas disease virus (SPDV, NC_003433, NC_003930); Seal louse virus (SESV, AF315122); Semliki Forest virus (SFV, NC_003215); Sindbis virus (SINV, NC_001547); Venezuelan equine encephalitis virus (VEEV, NC_001449); Western equine encephalitis virus (WEEV, NC_003908); Whataroa virus (WHAV, AF339479).

## 3. Results

In our study, a total of 81 full-length genomic RNA sequences of animal viruses were analyzed for the presence of potential H-type pseudoknots. To facilitate the analysis of such a large number of full-length sequences, in house-developed computer program is used, which is capable of identifying H-type pseudoknots efficiently and reliably. In brief, the program identifies pseudoknots by scanning through the input RNA sequence and testing every possible combination of stem and loop (S1, S2, L1, L2, and L3) lengths within the predefined ranges to see whether two helical stems can form simultaneously in a linear sequential topology as shown in Figure 1(a). This approach ensures that no potential pseudoknots with stem and loop lengths that fall within the predefined ranges would escape from being detected.

 In our pseudoknot search, we set the default ranges for stem and loop lengths as follows: stem1 (S1) and stem2 (S2) both have from 5 to 20 base pairs, loop1 (L1) has from 1 to 10 nucleotides, loop2 (L2) has from 3 to 50 nucleotides, and loop3 (L3) has from 0 (L3 is absent) to 10 nucleotides. These default ranges are very generous because most established H-type pseudoknots have stem and loop lengths that fall within these ranges. 

 To evaluate the relative strength of the identified pseudoknots in a given viral genomic RNA sequence, the free energy of the two stems for each of the identified pseudoknots was calculated, based on the Turner's nearest-neighbor parameters. The pseudoknots were ranked according to the calculated free energies. For a bioinformatic investigation, this calculated free energy represents the best way to evaluate the relative stabilities of the putative H-type pseudoknots within a given viral genome. The free energy value is also used as a criterion for the search; only those pseudoknots with a free energy value lower than −18 kcal/mol are kept for further analysis.

 In a typical search, tens of potential pseudoknots were identified within the full-lengths genomic viral mRNAs. For example, in the full-length genomic RNA of simian retroviruses type-1 (SRV-1, accession number M11841) that has 8173 nucleotides, 50 potential pseudoknots were identified using the default stem and loop ranges for pseudoknot formation. Some of these potential pseudoknots have overlapped pseudoknot-forming sequences; that is, two or more potential pseudoknots are mutually excluded and cannot exit at the same time. After eliminating overlapped pseudoknots with higher free energy, the number of potential pseudoknots in the SRV-1 genomic mRNA decreases to 31. Of course, it is possible that some of the detected pseudoknots may not really exist. Among the 31 detected pseudoknots, the established −1 frameshift stimulating pseudoknot at the *gag-pro* junction [25, 43] is identified as the most stable pseudoknot as judged by the lowest calculated free energy of −33.7 kcal/mol (Table 1). 

 While pseudoknots were detected shortly downstream from the frame-shift or read-through sites in most of the viral sequences using the default ranges of stem and loop lengths, the default search did miss some known cases, such as the frameshift stimulator pseudoknot in human coronavirus 229E that has a 164 nt L2. For such cases, the ranges of stem and loop lengths were increased accordingly for another round of search. At the end, possible pseudoknots were identified shortly downstream from the frame-shift or read-through sites in 69 full-length viral genomic mRNA sequences (85% of the 81 sequences). Table 1 lists information related to the detected frameshift- or readthrough-stimulating pseudoknots. This table does not include those viral mRNAs in which no pseudoknot was identified downstream from the slippery sequence. In Table 1, the viruses are grouped into different families and listed in the particular orders as in the ICTV (International Committee on Taxonomy of Viruses) 2011 Master Species List (MSL) version 2. 

 As documented in Recode V2.0: database of translational recoding events [42], a large number of viruses are known or expected to use a pseudoknot as the stimulator RNA structure for −1 frameshifting or readthrough. All but one (human astrovirus) of these documented pseudoknots are identified by our pseudoknot searching program. The putative frameshift stimulator pseudoknot in human astrovirus as shown in the Recode database has three mismatched pairs in a row within a five-basepair stem2, which explains why it is not detected by our program. Interestingly, the program identifies many potential frameshift stimulating pseudoknots in viruses whose frameshift stimulators are indicated as simple stem-loop structure (or absence of structure) in the Recode database. Below, we briefly describe the results.

**Figure 2:**
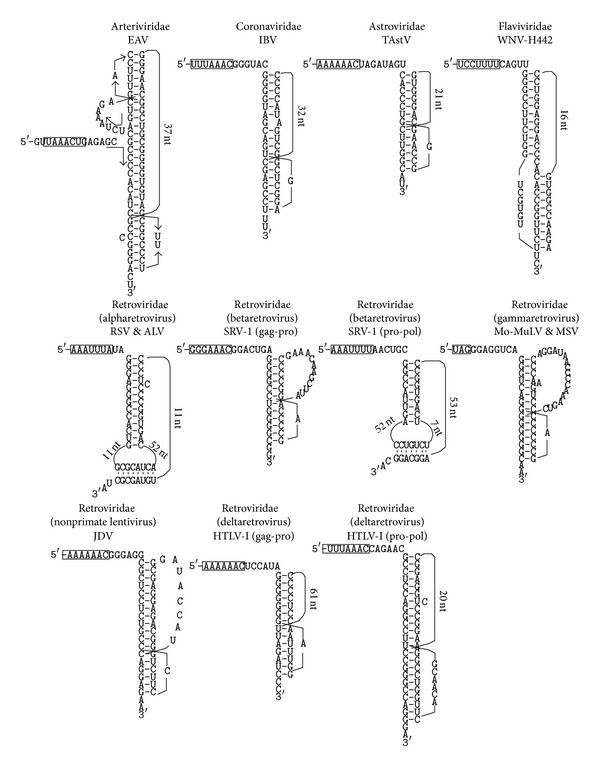
Representative frameshift or readthrough stimulator pseudoknots in different family of viruses. The slippery sequences or 0 frame stop condons are boxed.

*The Arteriviridae Family*. −1 frameshift stimulating pseudoknots identified in this family of viruses are pretty much identical to those shown in the Recode database (see Table 1 for summarized information of the pseudoknots and Figure 2 for schematic drawings of representative pseudoknots from various virus families). The involvement of the LV and LDV pseudoknots in efficient frameshifting was established [44, 45]. The pseudoknots in EAV, LV, LDV, and PRRSV are comparable to each other in terms of the lengths of the spacer region, S1, S2, L1, and L3, and the calculated stem free energies. The lengths of L2 are more varied. The length of L2 in EAV (68 nt) is substantially longer than those in other viruses of this family. Interestingly, we find that this L2 sequence harbours a potential pseudoknot with 31 nt (Figure 2). This pseudoknot seems very credible because it is very similar to the structurally well characterized T2 bacteriophage gene 32 mRNA autoregulatory pseudoknot [46] and the SRV-1 *gag-pro* frameshift stimulating pseudoknot [24, 25]. Moreover, this potential pseudoknot, when placed in an “up-side-down” orientation, stacks just right on top of the stem1 of the frameshifting pseudoknot. Stacking of the four stem regions of the two pseudoknots creates a quasi-continuous double helix of 28 basepairs in length. The slippery sequence in EAV (G UUA AAC) is somewhat deviated from the consensus slippery sequence of X XXY YYZ found in most other viruses. Whether the elaborate arrangement of pseudoknots in EAV plays any role in regulating frameshifting efficiency, and if it does, whether it is related to the “atypical” slippery sequence, await further investigations. All of the frameshift stimulating pseudoknots in EAV, LV, LDV, and PRRSV rank first among all of the potential pseudoknots identified within the full-length genomic RNAs. 

 The other virus of the Arteriviridae family, simian hemorrhagic fever virus (SHFV), also has a potential frameshift stimulating pseudoknot, which seems to be different from those pseudoknots in EAV, LV, LDV, and PRRSV. The potential frameshift stimulating pseudoknot in SHFV ranks 78th among all of the potential pseudoknots identified within the full-length genomic RNAs. The first ranked potential pseudoknot has a calculated stem free energy of −31.7 kcal/mol.

*The Coronaviridae Family*. PRF stimulating pseudoknots identified in this family of viruses at the ORF1a and ORF1b overlapping region are basically the same as those shown in the Recode database and a number of previous studies [18, 22, 47–50]. Most of these pseudoknots have comparable stems and loops (see Figure 2 for the IBV pseudoknot as a representative). All but one of these pseudoknots rank first in terms of calculated stem free energy among all of the potential pseudoknots identified within the full-length genomic RNAs. 

 The HCV229E frameshifting pseudoknot has a long (164 nt) L2. It was found that a short stretch of nucleotides at the 3′-end of L2 participated in the formation of an extra helical stem required for efficient frameshifting in HCV229E [49]. The extra stem has the potential to stack on stem S2 of the pseudoknot. The established frameshift stimulator pseudoknot in SARS also has an elaborated three stemmed structure [17, 18]. The 5′-end sequence of L2 has the potential to form a stem-loop structure. The extra stem has the potential to stack on stem S1 of the pseudoknot. 

The frameshift stimulator pseudoknot in PEDV is different from those pseudoknots in other viruses of this family. Most noticeably, the length of S1 is much shorter (5 bp versus 11–14 bp). Correspondingly, the pseudoknots are less stable. It ranks beyond 100th among all the potential pseudoknots within the gemones. The 1st ranked putative pseudoknot in this virus has a calculated free energy of −38.7 kcal/mol. 

*The Astroviridae Family*. There are five astroviruses (human, ovine, mink, turkey and chicken astroviruses) in the Recode database. All viruses use the same slippery sequence A AAA AAC. According to the Recode database, human astrovirus and chicken astrovirus use a pseudoknot and a stem-loop as the frameshift stimulator, respectively, while there is no information on the other three viruses. However, we detected potential pseudoknots 6–8 nt downstream from the slippery sequences in ovine, turkey and chicken astroviruses. The detected pseudoknots have comparable stem and loop lengths, as well as stem free energies. They rank 2nd, 10th, and 16th among all the potential pseudoknots within the gemones.

*The Flaviviridae Family*. Pseudoknots detected in this family are identical to those documented in the Recode database. Two slippery sequences are used by these viruses: C CCU UUU and U CCU UUU. The pseudoknots are very similar to each other in terms of the lengths of the stems & loops and stability. In all but one of the viruses, the putative frameshift stimulating pseudoknot rank 1st among all potential pseudoknots within the genome. The frameshift stimulating pseudoknot in Murray Valley encephalitis virus ranks 2nd. The 1st ranked pseudoknot has a calculated stem free energy of −38.9 kcal/mol, which is slightly lower than the frameshift stimulating pseudoknot.

*The Retroviridae Family*. Viruses in the retroviridae family belong to several genera: alpha-, beta-, gamma-, delta-, or epsilon-retroviruses and lentivirus. These retroviruses utilize three different mechanisms to express their *gag*, *pro* and *pol* genes from a single *gag-pro-pol *translational unit: (1) in-frame readthrough of the *gag* termination codon (gamma- and epsilon-retroviruses); (2) single frameshift event at the *gag-pol* junction to express the *pol *gene (alpharetrovirus and lentivirus); (3) double frameshift events at the *gag-pro* and *pro-pol* junctions to express the *pro* and *pol* genes (beta- and delta- retroviruses). 

 The two alpharetroviruses Avian leukosis virus (ALV) and Rous sarcoma virus (RSV) have very similar sequences at the *gag-pol* frameshift junction and both have a pseudoknot. The pseudoknot in RSV as a frameshift stimulator has been established [51]. The ALV and RSV pseudoknots contain a very long L3 (52 nt) which is much longer than those in most other known frameshift stimulator pseudoknots. Due to the unusual length of L3 (not within the default range), the ALV and RSV frameshift stimulator pseudoknots initially were not detected. They were detected after we increased the upper limit for L3 to 60 nt. Both pseudoknots ranked 1st among all possible pseudoknots within the viral genomes. The RSV and ALV pseudoknots as shown in Table 1 leave only one nucleotide in the spacer, which is too short to position the slippery sequence at the active site of the ribosome while leaving the pseudoknot at the entrance of the mRNA tunnel based on model building studies [13, 52]. However, this problem can be solved easily by breaking an appropriate number of base-pairs within stem1 adjacent to the spacer.

 Many betaretroviruses were investigated (JSRV to mIAP in Table 1). All of the viruses in this group rely on double frameshifting mechanism to express their *pro* and *pol* genes. Table 1 shows the detected pseudoknots associated with *gag-pro* frameshifting (potential *pro-pol* frameshift stimulator pseudoknots are not listed in Table 1). Most of these pseudoknots are identical to those previously reported and shown in the Recode database. These pseudoknots are comparable to each other in terms of the lengths of the stems and loops. They are all very compact pseudoknots with less than 35 nt. L3 is absent in all but one (MMTV) of the pseudoknots. All pseudoknots ranked 1st among all possible pseudoknots within the viral genomes (using the default ranges of stem and loop lengths in the search). 

 No pseudoknot was detected downstream from the *pro-pol* frameshift site in any of the betaretroviruses when the default ranges for stem and loop lengths were used in the search. However, when we increased the upper limit of L1 to 60 nt and performed another round of search, potential frameshift stimulator pseudoknots were detected in all viruses (For an example pseudoknot, see Figure 2. See Supplementary Figure 1 for all of the potential pseudoknots). These pseudoknots are much bigger than the compact *gag-pro* frameshift stimulator pseudoknots. They all have a relatively large L1 (ranging from 36 to 52 nt) and L2 (ranging from 15 to 52 nt); the total numbers of basepairs in the two stems are also larger (ranging from 14 to 20 bp). The functional importance of the *gag-pro* frameshift pseudoknots in the betaretroviruses has been well established [24, 25, 53–55]. In contrast, the utilization of a pseudoknot as the frameshift stimulator for *pro-pol *frameshifting has not been established.

 Four deltaretroviruses (BLV to STLV in Table 1) were investigated. These viruses also utilize double frameshift mechanisms to express their *pro* and *pol* genes. The secondary RNA structures downstream of the *gag-pro* frameshift sites in this group of retroviruses were generally believed to be simple stem-loops [22, 56, 57]. Using the default ranges for stems and loops, potential pseudoknots were detected downstream from the *gag-pro* frameshift site in all viruses but BLV (not shown). However, credibility of the detected pseudoknots is questionable due to the lack of appropriate spacer between the slippery sequence and the pseudoknot. In view of the fact that the previously reported stem-loop structures seem to be conserved in all four viruses, we increased the upper limit for L2 to 85 nt and performed another search. Interestingly, potential pseudoknots with decent stability were detected downstream from the *gag-pro* frameshift site in all four viruses (shown in Table 1); moreover, the detected pseudoknots are all formed by basepairing of a stretch of nucleotides in the loop of the previously reported stem-loop structure with a complimentary sequence 61–83 nt downstream. 

 Very stable pseudoknots were also detected downstream from the *pro-pol* frameshift site in all four deltaretroviruses (For an example pseudoknot, see Figure 2. See Supplementary Figure 1 for all of the potential pseudoknots). Similar to the SRV group, the putative *pro-pol* frameshift stimulator pseudoknots are bigger than the *gag-pro* pseudoknots. The *pro-pol* pseudoknots in HTLV-I and STLV-I are the most stable pseudoknots among all of the detected pseudoknots in this study (Figure 2), with a calculated free energy of −54.2 kcal/mol.

 Gammaretroviruses and epsilonretroviruses utilize the in-frame read-through decoding mechanism. For the two epsilonretroviruses Walleye dermal sarcoma virus and Snakehead retrovirus, no pseudoknot was detected downstream from the *gag* reading frame stop codon. The most stable pseudoknots detected in these two viruses have calculated free energy values of −33.0 and −40.7 kcal/mol respectively. For the gammaretroviruses (indicated by “RT” in the “SS or RT” column in Table 1), conserved pseudoknots were detected downstream from the *gag* reading frame stop codon in all viruses. These pseudoknots are the same as previously proposed [22] and shown in the Recode database. The indispensable role of the Mo-MuLV pseudoknot in readthrough suppression had been established by two independent studies [20, 21]. All of these putative pseudoknots ranked 1st or 2nd among all possible pseudoknots within the viral genome. 

 The lentiviruses investigated include several non-primate lentiviruses (BIV to PLV-14 in Table 1) and three primate lentiviruses (HIV-1, HIV-2 and SIV). Potential pseudoknots were detected downstream from the *gag-pol* frameshift site in all non-primate lentiviruses. These frameshift stimulator pseudoknots are largely the same as previously reported and documented in the Recode database [22, 58–60]. All but two of these pseudoknots ranked 1st among all possible pseudoknots within the viral genome. 

 There are three primate (simian or human) lentiviruses: simian immunodeficiency viruses (SIV) and human immunodeficiency viruses type-1 and type-2 (HIV-1 and HIV-2) in the Recode database. The database gives only one representative sequence for each of these viruses. These are the particular sequences we investigate in this study. No potential pseudoknot was detected downstream from the *gag-pol* frameshift site in SIV. 

**Figure 3:**
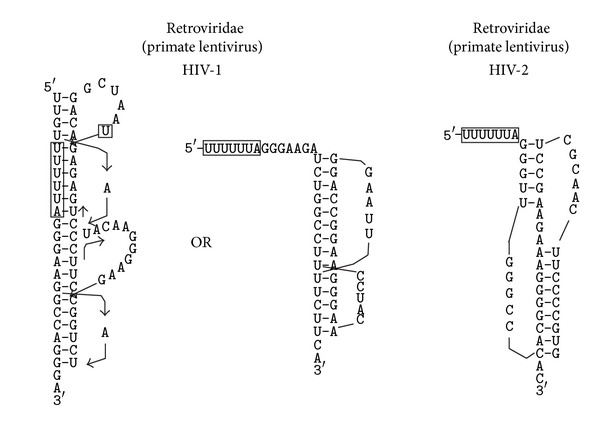
Detected pseudoknots at the *gag-pol* frameshift junction of HIV-1 and HIV-2. The slippery sequences are boxed.

 In HIV-1 (strain HXB2), the sequence downstream from the *gag-pol* frameshift site harbours two potential pseudoknots that are mutually excluded (Figure 3). One of the potential pseudoknots is preceded by a normal length spacer, while the other potential pseudoknot follows the slippery sequence by 0 or 1 nucleotides (depending on whether a G-U basepair is formed in S1). The two pseudoknots rank 16th and 10th, respectively, among all potential pseudoknots within the genomic mRNA (only the 10th ranked pseudoknot is listed in Table 1). The 1st ranked potential pseudoknot has a calculated free energy of −29.6 kcal/mol. Intriguingly, we detected another potential pseudoknot that involves the slippery sequence U UUU UUA (boxed in Figure 3). The two potential pseudoknots in the *ga-pol* junction region have the potential to stack their stems together to form a quasicontinuous double helix with 22 basepairs. Interestingly, the two potential pseudoknots are very similar to the established SRV-1 *gag-pro* frameshift stimulator pseudoknot. They are all compact pseudoknots belonging to the previously proposed CPK-1 (standing for common pseudoknot motif 1) type [46, 61] (more details in Section 4). 

 In HIV-2, a compact pseudoknot was detected immediately downstream from the *gag-pol* frameshift site (Figure 3). This potential pseudoknot ranks 5th among all potential pseudoknots within the genomic mRNA. The 1st ranked potential pseudoknot has a calculated free energy of −30.5 kcal/mol.

**Figure 4:**
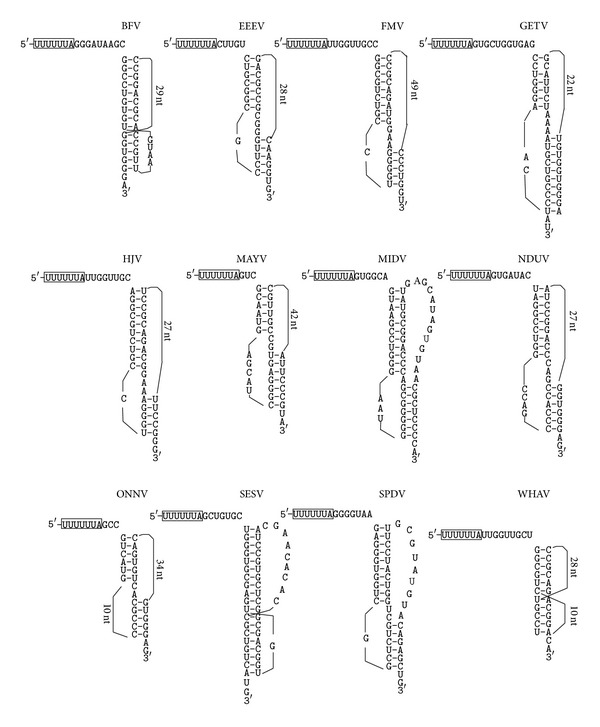
Detected putative frameshift stimulator pseudoknots in the Togaviridae family. The slippery sequences are boxed.

*The Togaviridae Family*. Twenty viruses in this family (all belonging to the alphavirus genus) were investigated. For three of these viruses (MIDV, NDUV, and SESV), the Recode database predicts a pseudoknot structure downstream from the frameshift site. For the other viruses, the Recode database either predicts a simple stem-loop structure downstream from the frameshift site or makes no prediction. Using our pseudoknot search method, we detected putative frameshift stimulator pseudoknots in twelve viruses, including MIDV, NDUV and SESV (Table 1 and Figure 4). Half of these pseudoknots ranked 1st among all possible pseudoknots within the viral genomes. In those viruses in which no frameshift stimulator pseudoknot is detected or the detected frameshift stimulator pseudoknot does not rank 1st, no ultrastable pseudoknots are identified. The most stable possible pseudoknot detected in this group of viruses has a calculated free energy of −40.8 kcal/mol.

## 4. Discussion

We have used a robust in house-developed computer program to detect potential pseudoknots within the full-length genomic mRNAs of a large number of viruses. In many of these viruses, a frameshift or readthrough stimulator pseudoknot was verified or predicted previously (as documented in the Recode database). All but one of these pseudoknots were detected by our program. The missed case is in human astrovirus, in which the predicted framshift stimulator pseudoknot by the Recode database is very weak. Importantly, our approach of pseudoknot detection was not restricted to a limited sequence window downstream from the known frameshift or readthrough recoding sites. Instead, the program detects all possible pseudoknots within the full-length viral genomic mRNAs. The effectiveness and reliability of our approach are proven by the fact that almost all of the previously documented frameshift or readthrough stimulator pseudoknots are detected. 

 Interestingly, we also detected quite a number of putative frameshift stimulator pseudoknots that were not known before. Overall, potential pseudoknots were detected downstream from most (~90%) of the established or putative frameshift or readthrough sites (the *gag-pro* and *pro-pol* sites in the same virus are counted as two different sites). Some of these detected pseudoknots may not actually exist. However, the high percentage of possible pseudoknots detected downstream from the strategically important frameshift or readthrough sites still overwhelmingly proves that pseudoknots are the most common stimulators for efficient −1 ribosomal frameshifting and readthrough. 

 Since all possible pseudoknots within the full-length viral genomic mRNAs are detected in a blind search, the results from this study provide a new way to assess the significance and uniqueness of the frameshift or readthrough stimulator pseudoknots in an unbiased manner. As shown in Table 1, in ~78% of the viruses, the pseudoknot detected downstream from the frameshift or readthrough site rank 1st or 2nd among all possible pseudoknots within the genome. The pseudoknot with the lowest free energy (−48.7 kcal/mol) in Table 1 is found in Lelystad virus. The detected pseudoknot downstream from the *pro-pol* framshift site in HTLV-I/STLV-I (Figure 2, not listed in Table 1) has an even lower free energy of −54.2 kcal/mol. This pseudoknot is the most stable pseudoknot among all the possible pseudoknots (regardless of locations of the pseudoknots within the viral genomes) detected in this study. In comparison, the artificial strong pseudoknots that can act as ribosomal roadblocks described in a previous study [40] has a calculated free energy of −73.9 kcal/mol (22 bp in S1 and 6 bp in S2). Apparently, the frameshift or readthrough stimulator pseudoknots in a lot of viruses have evolved to become the most stable pseudoknot within the viral genomic mRNAs; but at the same time they are not too strong. These pseudoknots seem to have optimal stability to stimulate the right amount of frameshifting and readthrough and subsequently be unfolded by the translating ribosomes. In other viruses in which the detected frameshift or readthrough stimulator pseudoknot ranks lower or no pseudoknot is detected downstream from the frameshift site, the most stable potential pseudoknots all have a calculated free energy value higher than the putative *pro-pol* framshift stimulator pseudoknot in HTLV-I/STLV-I. These results clearly show that the viral genomic mRNAs do not contain ultra-stable “roadblocking” pseudoknots that would significantly stall ribosomes and might induce no-go decay of the mRNAs [40, 62]. 

 It was noticed previously that many naturally occurring pseudoknots (not limited to frameshift and readthrough stimulating pseudoknots) belonged to a structurally related pseudoknot family known as CPK-1, standing for common pseudoknot motif 1 [46, 61]. A typical CPK-1 pseudoknot has a S2 of 6-7 base pairs and a very short L1 of 1-2 nucleotides; L3 is absent therefore the two helical stems S1 and S2 can stack to form a quasicontinuous helix. An inspection of the detected frameshift or readthrough stimulator pseudoknots reveals that more than 40% of these pseudoknots conform to the CPK-1 family (Table 1). The alternative tandem pseudoknots (Figure 3. Not listed in Table 1) in HIV1 and the elaborated pseudoknot in EAV (Figure 2) also conform to the CPK-1 family. Interestingly, it was found that the founding member of the CPK-1 family, a pseudoknot in gene 32 mRNA of bacteriophage T2 whose natural biological function is translational autoregulation, was unable to serve as a frameshift stimulator [63]. Most likely, the common features defined by the CPK-1 motif may primarily serve a structural role for maintaining a stable and compact pseudoknotted scaffold upon which diverse biological functions can build on, presumably mainly by the more variable parts of the pseudoknots, including S1 & L2, and possible interactions between them.

Consistent with this theory, it was found in several different systems that frameshifting efficiency was generally more sensitive to mutations introduced to S1 & L2, and the junction, while mutations to S2 & L1 showed less effect on frameshifting efficiency, as long as integrity and stability of the pseudoknot-forming interaction were maintained [25, 63–66]. This theory provides a very good explanation for the frequent utilization of CPK-1 type pseudoknots in various viruses. It is clear that while a large number of detected pseudoknots conform to the CPK-1 family, the lengths and compositions of S1 and L2 of these pseudoknots show a fair degree of variations, especially when viruses from different groups are compared. Moreover, additional features could be added to the “basic” CPK-1 pseudoknot fold (such as seen in the EAV, HCV229E and *SARS *pseudoknots) which would make the pseudoknots even more versatile in fine-tuning the frameshifting.

 Although CPK-1 type pseudoknots occur most frequently among the detected pseudoknots, other types of pseudoknots are also observed. These pseudoknots sample a wide range of stem and loop sizes, as well as the presence or absence of an intervening sequence (L3). Given the wide range of sequences studied, it is not particularly surprising to observe all these variations associated with the detected pseudoknots. Since different viruses (especially viruses that are remote in evolution) may have different requirements for certain level of frameshifting efficiency, variations in the frameshifting pseudoknots, as well as the slippery sequences and spacers, may be necessary for the fine-tuning of the frameshifting efficiency to meet the specific need of different viruses. 

 The frameshift stimulating secondary structure downstream of the HIV-1 group M (which includes the strain HXB2 investigated in this study) *gag-pol *frameshift site was originally proposed to be a simple stem-loop [67], which was shown to be important for wild-type level frameshifting *in vivo* (in mammalian cells) [68]. It was also shown that a sequence downstream from the originally proposed stem-loop also contributed to frameshifting, either modelled as an intramolecular triplex [69] or an extended bulged stem-loop [70]. In HIV-1 strain MVP5180 from subgroup O, a very classic H-type pseudoknot locating 8nt downstream from the *gag-pol* frameshift site was shown to be required for stimulating a higher frameshifting efficiency than that in group M [71]. 

 Our study detected potential pseudoknots in the *gag-pol* junction of both HIV-1 and HIV-2. In HIV-1 (HXB2), two mutually excluded potential pseudoknots were detected downstream from the slippery sequence (Figure 3), one with no spacer and the other with a normal length spacer. Intriguingly, another pseudoknot that contains the slippery sequence is detected. This pseudoknot can stack on top of the pseudoknot immediately downstream from the slippery sequence. Given such an elaborated arrangement of tandem pseudoknots (both belonging to the CPK-1 family) and another mutually excluded pseudoknot at the HIV-1 *gag-pol* frameshift junction, we asked ourselves this question: can we come up with a reasonable hypothesis about the PRF mechanisms in this case that would explain the possible involvement of these pseudoknots? 

 The answer is: yes we can. Let us assume that the tandem pseudoknots are present at the *gag-pol* frameshift junction (due to its lower free energy compared to the alternative pseudoknot). These pseudoknots can significantly slow down the translating ribosome when the pseudoknots are being unwound by the ribosome. When the ribosome scans through the unwound mRNA sequence and is approaching the slippery sequence, the top pseudoknot is fully unwound and the stem1 of the bottom pseudoknot should also be disrupted. Stem2 of the bottom pseudoknot remains intact. The six basepairs of this stem2 are actually the same as the first six basepairs in stem1 of the mutually excluded pseudoknot (Figure 3). Now that the tandem pseudoknots are unwound, the alternative pseudoknot can form rapidly (because a large portion of its stem1 is already in place). The newly formed pseudoknot, with an optimal spacer from the slippery sequence, jams the entrance of the mRNA tunnel of the ribosome. This novel mechanism of PRF elegantly explains the results from our bioinformatic study and is consistent with current paradigm of PRF mechanism. Equilibrium of relevant alternative RNA structures has been shown to play a functional role in the regulation of read-through efficiency in murine leukaemia virus, suggesting a general involvement of equilibrium-based mechanism in translational recoding [72]. We plan to carry out a large scale analysis on the several thousand sequences for different strains of HIV1 viruses to assess the degree of conservation on the putative PRF signals.

## 5. Conclusion

Several conclusions can be drawn from our study. At first, the viral genomic mRNAs do not contain strong roadblocking pseudoknots that would terminal translation. Second, the frameshift or readthrough stimulating pseudoknots in most viruses are among the most stable pseudoknots within the viral genomic mRNAs. The stabilities of these pseudoknots have been fine-tuned during evolution to be optimal for the decoding events. Third, pseudoknots of the CPK-1 family occur most frequently. The favorable CPK-1 scaffold can accommodate significant variations (especially in the stem1 and loop2 regions) which are presumably important for the fine-tuning of framshift or readthrough stimulating ability of the pseudoknots. Fourth, some HIV1 viruses may utilize a novel mechanism that involves three pseudoknots to regulate the frameshift efficiency at the *gag-pol* junction. Results from this study also prove the usefulness of our pseudoknot-detecting program. Since this is a general-purpose program that can identify all possible pseudoknots in a long RNA sequence, we expect that the program will find its application in some other related studies such as identifying potential cases of pseudoknot-dependent −1 PRF in cellular genes.

## Supplementary Material

In a computer-generated output (figure below), the sequence elements of the detected pseudoknot (See Figure 1 of the article for sequence elements of a pseudoknot) and a sequence of 20 nt preceding the pseudoknot are shown in three lines. The first line is for L2 and L1. The second line is for S1-3' and S2-5' sequences. The third line is for the 20 nt preceding sequence (which may contain the slippery sequence and spacer region), S1-5', and S2-3' sequences. Note that the 5'→3' orientation of the sequence in the second line runs from the right to the left, which is opposite to the sequences in the other two lines. The free energy (∆Go37) values for the two stems S1 and S2 are calculated and indicated. The output also indicates the starting/ending positions of the pseudoknot-forming sequence, the number of nucleotides in the stems/loops (S1, S2, L1, L2, and L3) of the pseudoknot. The detected pseudoknots in a viral genome are ranked by the calculated free energy values.
In the sample output, some pseudoknots have their pseudoknot-forming sequences overlapped (such as the first and second ranked pseudoknots), meaning that the pseudoknots cannot exist simultaneously. The overlapped pseudoknots with a higher free energy are indicated by a line through the middle of the selected text.

## Figures and Tables

**Table 1:** Detected pseudoknots downstream from the frameshift site (slippery sequence) or 0 frame stop codon in viruses.

Virus	SS or RT	sp	S1	S2	L1	L2	L3	Δ*G*	Rank	CPK1
Artiriviridae										
Equine arteritis virus (EAV)	GUUAAAC	5	10	7	2	58	0	43.3	1	Yes
Lelystad virus (LV)	UUUAAAC	5	12	7	4	15	0	48.7	1	
Lactate dehydrogenase-elevating virus (LDV)	UUUAAAC	6	11	8	3	17	0	41.7	1	
Porcine reproductive and respiratory syndrome virus (PRRSV)	UUUAAAC	4	13	7	4	21	0	47.7	1	
Simian hemorrhagic fever virus (SHFV)	UUUAAAC	7	5	7	8	9	1	21.3	78	

Coronaviridae										
Transmissible gastroenteritis virus (TGEV)	UUUAAAC	3	14	6	2	25	0	34.5	1	Yes
Bovine coronavirus (BCV)	UUUAAAC	5	11	9	2	32	2	38.8	1	
Human coronavirus 229E (HCoV-229E)	UUUAAAC	5	12	7	1	164	0	39.0	1	Yes
Human coronavirus OC43 (HCoV-OC43)	UUUAAAC	5	11	9	2	32	2	38.7	1	
Porcine epidemic diarrhea virus (PEDV)	UUUAAAC	7	5	6	1	18	0	19.8	>100	Yes
Human coronavirus HKU1 (HCoV-HKU1)	UUUAAAC	5	12	9	1	32	1	39.0	1	
Murine hepatitis coronavirus (MHV)	UUUAAAC	5	13	7	1	32	0	37.1	1	Yes
SARS coronavirus (SARS-CoV)	UUUAAAC	5	11	7	1	32	0	28.7	1	Yes
Avian infectious bronchitis coronavirus (IBV)	UUUAAAC	6	11	7	1	32	0	35.6	1	Yes
Bovine torovirus (Breda virus) (BRV)	UUUAAAC	5	11	6	1	11	2	37.1	1	
Equine torovirus (Berne Virus) (BEV)	UUUAAAC	5	11	6	2	10	0	37.3	1	Yes

Astroviridae										
Chicken astrovirus (CAstV)	AAAAAAC	6	5	5	3	11	0	22.1	10	
Turkey astrovirus (TAstV)	AAAAAAC	8	7	6	1	21	0	30.4	2	Yes
Ovine astrovirus (OAstV)	AAAAAAC	6	5	5	8	20	0	26.2	16	

Flaviviridae										
Japanese encephalitis virus (JEV)	CCCUUUU	5	11	7	7	16	2	39.2	1	
Murray Valley encephalitis virus-Alfuy (ALFV)	CCCUUUU	5	11	6	8	16	2	37.3	2	
Murray Valley encephalitis virus (MVEV)	UCCUUUU	5	11	8	6	40	2	29.9	1	
Usutu virus (USUV)	UCCUUUU	5	11	7	7	16	2	39.2	1	
West Nile virus-Kunjin (WNVKUN)	UCCUUUU	5	11	7	6	17	3	37.6	1	
West Nile virus H442 (WNV-H442)	UCCUUUU	5	11	8	6	16	2	40.9	1	
West Nile virus (WNV)	CCCUUUU	4	11	7	6	17	3	36.9	1	

Retroviridae										
Avian leukosis virus (ALV)	AAATTTA	2	13	8	11	11	52	35.8	1	
Rous sarcoma virus (RSV)	AAATTTA	2	13	8	11	11	52	36.2	1	
Jaagsiekte sheep retrovirus (JSRV)	GGGAAAC	7	6	6	1	11	0	33.2	1	Yes
Mason-Pfizer monkey virus (MPMV)	GGGAAAC	7	6	6	1	12	0	33.7	1	Yes
Simian retrovirus 2 (SRV-2)	GGGAAAC	7	6	6	1	12	0	33.7	1	Yes
Simian retrovirus 1 (SRV-1)	GGGAAAC	7	6	6	1	12	0	33.7	1	Yes
Mouse mammary tumor virus (MMTV)	AAAAAAC	7	5	7	1	8	1	29.7	1	
Squirrel monkey retrovirus (SMRV)	GGGAAAC	7	6	7	1	14	0	33.8	1	Yes
Human endogenous retrovirus K10 (HERV K10)	GGGAAAC	7	6	6	1	9	0	31.8	1	Yes
Intracisternal A particle, Syrian hamster (IAP-H18)	AAAAAAC	7	7	7	2	7	0	37.7	1	Yes
Intracisternal A particle, Chinese hamster (CHIAP34)	AAAAAAC	7	7	7	2	8	0	37.9	1	Yes
Intracisternal A particle, mouse (m-IAP)	AAAAAAC	8	7	6	1	9	0	33.9	1	Yes
Bovine leukemia virus (BLV)	AAAAAAC	7	6	6	2	63	0	22.4	26	Yes
Primate T-lymphotropic virus 1 (HTLV-I)	AAAAAAC	6	9	6	1	57	0	32.7	1	Yes
Primate T-lymphotropic virus 2 (HTLV-II)	AAAAAAC	7	8	6	1	83	0	28.6	3	
Primate T-lymphotropic virus 3 (STLV-I)	AAAAAAC	6	9	6	1	57	0	35.4	1	Yes
Baboon endogenous virus (BaEV)	RT	8	9	6	1	19	0	38.1	1	Yes
Feline leukemia virus (FeLV)	RT	8	8	6	2	18	0	37.2	2	Yes
Gibbon ape leukemia virus (GaLV)	RT	8	8	7	2	17	0	37.0	1	Yes
Moloney murine sarcoma virus (MSV)	RT	8	8	7	1	18	0	40.9	2	Yes
Moloney murine leukemia virus (Mo-MuLV)	RT	8	8	7	1	18	0	40.9	2	Yes
Bovine immunodeficiency virus (BIV)	AAAAAAC	7	11	6	1	29	0	34.4	1	Yes
Jembrana disease virus (JDV)	AAAAAAC	6	12	6	1	8	0	40.8	1	Yes
South African Ovine Maedi Visna virus (SA-OMVV)	GGGAAAC	7	6	6	1	12	0	40.2	1	Yes
Caprine Arthritis Encephalitis virus (CAEV)	GGGAAAC	4	10	7	1	15	0	38.7	1	Yes
Equine infectious anemia virus (EIAV)	AAAAAAC	9	5	5	4	9	0	20.2	42	
Feline immunodeficiency virus (FIV)	GGGAAAC	6	7	6	2	9	0	33.9	1	Yes
Puma lentivirus (PLV)	AAAAAAC	8	5	6	2	11	0	24.6	12	Yes
Human immunodeficiency virus 1 (HIV1-HXB2)	UUUUUUA	7	9	5	5	5	0	23.5	10	
Human immunodeficiency virus 2 (HIV2)	UUUUUUA	0	5	9	5	6	3	29.6	5	

Togaviridae										
Barmah Forest virus (BFV)	UUUUUUA	9	9	5	4	29	0	26.7	6	
Eastern equine encephalitis virus (EEEV)	UUUUUUA	5	7	5	1	28	3	25.7	8	
Fort Morgan virus (FMV)	UUUUUUA	9	9	5	1	49	3	27.9	1	
Getah virus (GETV)	UUUUUUA	11	6	10	2	22	3	19.7	26	
Highlands J virus (HJV)	UUUUUUA	8	10	5	1	27	3	32.3	1	
Mayaro virus (MAYV)	UUUUUUA	3	6	7	5	42	2	23.3	34	
Middelburg virus (MIDV)	UUUUUUA	6	10	7	3	13	1	41.0	1	
Ndumu virus (NDUV)	UUUUUUA	7	9	6	4	27	3	36.1	1	
O'nyong-nyong virus (ONNV)	UUUUUUA	3	6	5	10	34	2	19.9	57	
Seal louse virus (SESV)	UUUUUUA	7	11	7	2	9	0	38.0	1	Yes
Salmon pancreas disease virus (SPDV)	UUUUUUA	8	9	6	1	10	2	25.8	27	
Whataroa virus (WHAV)	UUUUUUA	9	6	5	10	28	0	24.6	1	

SS or RT: slippery sequence or readthrough of stop codon; sp: length of the spacer sequence between the slippery sequence/stop codon and the downstream pseudoknot; S1, S2, L1, L2, and L3: lengths of the sequence elements of the pseudoknot, stem1, stem2, loop1, loop2, and loop3. See Figure 1 for the sequence elements of a typical pseudoknot. Calculated free energy of the stem regions of the pseudoknot is listed in the column “Δ*G*” in minus kcal/mol. “Rank” indicates the relative ranking (according to the calculated free energy of the stems) of the frameshift/readthrough stimulating pseudoknots among all possible pseudoknots detected within the full-length genomic RNAs. “CPK1” indicates whether the pseudoknot belongs to the CPK1 family.
