# Supplementary material for: A Genome-Wide Analysis of RNA Pseudoknots That Stimulate Efficient −1 Ribosomal Frameshifting or Readthrough in Animal Viruses
Source: Biomed Res Int. 2013 Nov 4;2013:984028. doi: 10.1155/2013/984028 (PMC3835772; doi:10.1155/2013/984028)

```
Sequence #: M11841 (8173) nucleotides

Energy 1: -34.710000 (kcal/mol): Start=2337 S1=6 S2=6 L1=1 L2=12 L3=0 End=2373
                    GAAACAAGCTTA       A
                    CCCCGG    ACCCCG
CCATCAGGGAAACGGACTGAGGGGCC    TGGGGCGG

Energy 2: -24.750000 (kcal/mol): Start=2351 S1=6 S2=6 L1=4 L2=39 L3=1 End=2417
                    GGTCAGCTTTGTTCCAGCCAACAAAAACAACCCATTTCA       AAAC
                    CGGGGT  A  TTCGAA
GACTGAGGGGCCAGCCCCAGGCCCCG     AAGCTTAC

Energy 3: -23.680000 (kcal/mol): Start=3480 S1=5 S2=6 L1=4 L2=36 L3=0 End=3541
                    ATTAATGGGAGCTTTACAACCTGGATTGCCCTCTCC       TAAT
                    TGGTA    TCACCG
GGCTCTTACAAGATTTACGAGCCGT    GGTGGCTA

Energy 4: -23.325000 (kcal/mol): Start=1502 S1=7 S2=5 L1=1 L2=11 L3=0 End=1537
                    CACATTAGCCA       C
                    ATACCCC    GTCAT
TAAAAACGGCTGCCTCTCAATATGGGG    TAGTAGA

Energy 5: -23.280000 (kcal/mol): Start=7255 S1=6 S2=6 L1=1 L2=29 L3=1 End=7308
                    GAGGCATCTGCTTAGCTTTACAGGAAAAA       G
                    GGACGA  G  ACGACA
CAAAACAGAAGAGGATTAGATCTGCT     TGCTGTTT

Energy 6: -23.010000 (kcal/mol): Start=6464 S1=5 S2=7 L1=6 L2=15 L3=2 End=6508
                    CTTTGCCCGAAGCCC       ACTTTC
                    GGTCT  CC  CACCATT
AATTGCATAAATCGCTGTTCCCAGA      GTGGTAAAG

Energy 7: -22.910000 (kcal/mol): Start=3444 S1=6 S2=10 L1=8 L2=29 L3=2 End=3512
                    ACGAGCCGTTAATGCCACTATGGTATTAA       GGTAAATG
                    TTTAGA  AC  ATTCTCGGAG
CCATATTTGTTATAAAAAAGAAATCT      TGGGAGCTTTAC
```

~~Energy 8: -22.750000 (kcal/mol): Start=7264 S1=6 S2=7 L1=4 L2=20 L3=0 End=7313~~

~~TAGCTTTACAGGAAAAATGC   GCAG~~

~~TCGTCT   ACGGAGG~~

~~AGAGGATTAGATCTGCTGACAGCAGA   TGTTTCTAC~~

Energy 9: -22.370000 (kcal/mol): Start=1310 S1=5 S2=6 L1=8 L2=27 L3=0 End=1366

CAACATATCCCTAAAGGAAAATGCTGC   CACACCTC

GGGGC   CGGTCA

CAGAAACTGCAGGGGGCTTTTCTCG   GCCAGTCG

~~Energy 10: -22.140000 (kcal/mol): Start=3520 S1=5 S2=6 L1=1 L2=13 L3=1 End=3555~~

~~TGGCTATCCCACA   T~~

~~GGCCT C TCCCGT~~

~~ATTAATGGGAGCTTTACAACCTGGA   AGGGTATC~~

Energy 11: -22.025000 (kcal/mol): Start=6081 S1=5 S2=8 L1=2 L2=18 L3=0 End=6126

CCCACTACAGCCAGCCCC   CA

TCATC   TGTGTGTG

TTCAGTAACCAACTCCCTAAAGTGG   ACACATATAG

Energy 12: -21.995000 (kcal/mol): Start=6588 S1=6 S2=6 L1=2 L2=5 L3=0 End=6618

TGCTT   AA

GTCGGT   CGTTAG

TGTTTCCTCCCAACGCCAATTAGCCG   GCGGTCAG

Energy 13: -21.485000 (kcal/mol): Start=2683 S1=5 S2=8 L1=2 L2=8 L3=0 End=2718

CCTACCTC   AG

TCCTA   ATTAACTC

TTACTGTTCCTCAAGGCAACAGGAT   TAATTGAGAC

~~Energy 14: -21.235000 (kcal/mol): Start=2690 S1=7 S2=5 L1=10 L2=27 L3=0 End=2750~~

~~ACAGACAATAAAGTACAACAACCCTAT   AATCCTCCTA~~

~~GAGTTAA   TCTCC~~

~~TCCTCAAGGCAACAGGATAGCTCAATT   AGAGGAC~~

```
Energy 15: -21.215000 (kcal/mol): Start=1744 S1=5 S2=6 L1=4 L2=30 L3=0 End=1799
                    TTGCTCAAATTCAAGCGGCTGCTACAAAAG       ATGA
                    TGTTA    GGGCCT
TCTAGCACTGATGCACAAATGCAAT    CCTGGAGA

Energy 16: -21.005000 (kcal/mol): Start=206 S1=5 S2=8 L1=2 L2=33 L3=0 End=266
                    TTGCCACCATTAAACGAGACTTGATCAGAACAC       AT
                    ACGCA    ACAGAATA
CTCCCCTCCCACTTACTGCCTGTGT    TGTCTTGTCT

Energy 17: -21.005000 (kcal/mol): Start=8029 S1=5 S2=8 L1=2 L2=33 L3=0 End=8089
                    TTGCCACCATTAAACGAGACTTGATCAGAACAC       AT
                    ACGCA    ACAGAATA
CTCCCCTCCCACTTACTGCCTGTGT    TGTCTTGTCT

Energy 18: -20.755000 (kcal/mol): Start=7256 S1=5 S2=5 L1=3 L2=8 L3=0 End=7286
                    GGAGGCAT      GAC
                    GACGA    GACGA
AAAACAGAAGAGGATTAGATCTGCT    CTGCTTA

Energy 19: -20.545000 (kcal/mol): Start=2762 S1=8 S2=5 L1=8 L2=47 L3=0 End=2842
                    CCTATTACCTGTCAGAAGCCCTCCTTAACATTATGGTTAGATGATAA       TCAGACAT
                    AACCTGGG    TTATA
CTATAGAGGACAAGGAAGTTTTGGATCC    AATGTTC

Energy 20: -20.520000 (kcal/mol): Start=6369 S1=8 S2=5 L1=4 L2=9 L3=0 End=6407
                    AGGGCCTCA       CACA
                    GGAGGTAG    TCTGT
GGTTTGCTGGAATAGCCAACCCTCTGTT    AGATAAG

Energy 21: -20.510000 (kcal/mol): Start=2905 S1=6 S2=7 L1=10 L2=3 L3=2 End=2943
                    TAA       CCTATAACAG
                    TTAACC  AA  TTCCATA
AGCTAGAGGACTGGCCTCCTAATTGG      GAGGTATAG
```

Energy 22: -20.450000 (kcal/mol): Start=5810 S1=6 S2=5 L1=1 L2=36 L3=3 End=5868

AGATTGCCTTTTCCTTTGCTCACAGAGATGAACTTC G

GTCCCT CTC TTAGT

ATACCTAACAATAACCAATCCAGGGA AATCATC

Energy 23: -20.320000 (kcal/mol): Start=3314 S1=7 S2=6 L1=1 L2=12 L3=1 End=3352

AGTGAAAAACTT C

CCAATTA C CGGTGA

AAATCAGACGAACCTGTCTGGGTTGAT GCTGCTGC

Energy 24: -20.095000 (kcal/mol): Start=7088 S1=5 S2=5 L1=5 L2=3 L3=0 End=7115

TGG ATCTA

CGTCA GGGCC

TAGGATTAGGTATAACTACTGCAGT TCTGGGG

Energy 25: -20.000000 (kcal/mol): Start=2352 S1=5 S2=5 L1=4 L2=5 L3=2 End=2380

CGGTC AAAC

GGGGT AT TCGAA

ACTGAGGGGCCAGCCCCAGGCCCCG AGCTTTG

~~Energy 26: -19.895000 (kcal/mol): Start=2368 S1=7 S2=5 L1=3 L2=23 L3=0 End=2417~~

~~GCCAACAAAAACAACCCATTTCA GTC~~

~~ACCTTGT TTCGA~~

~~CAGGCCCCGAAACAAGCTTATGGGGCG AAGCTTA~~

Energy 27: -19.830000 (kcal/mol): Start=2228 S1=5 S2=5 L1=3 L2=45 L3=0 End=2295

TGTAAAAGAGGGAAACATTGGGCCAATGAATGCAAATCCAAAACT ACT

GGACC CTGTC

ACAATTCTGAAACAAAGGCTCCTGG GATAGTC

~~Energy 28: -19.760000 (kcal/mol): Start=1503 S1=5 S2=6 L1=1 L2=12 L3=1 End=1537~~

~~ACACATTAGCCA G~~

~~TACCC C GTCATC~~

~~AAAAACGGCTGCCTCTCAATATGGG TAGTAGAA~~

~~Energy 29: -19.550000 (kcal/mol): Start=2817 S1=6 S2=7 L1=7 L2=33 L3=1 End=2882~~

~~CACAGGATTAATCGATACGGGAGCTGATGTCAC ATGGTTA~~

~~TTGTAA A ATAGTAG~~

~~ACCTGTCAGAAGCCCTCCTTAACATT TATCATCAA~~

Energy 30: -19.550000 (kcal/mol): Start=4900 S1=5 S2=6 L1=8 L2=23 L3=1 End=4952

ACCTATAGCCCACGGCAACCAAA GCCCATTC

GGTCC A TCCGGT

ACCTTTTTACATCGGACATGTCAGG AGGCTGAC

Energy 31: -19.360000 (kcal/mol): Start=728 S1=6 S2=5 L1=5 L2=29 L3=2 End=783

AATTTAATTAAAGAATTGATAGATAAGAA ACTGC

GGTCAT AC TCTTC

CTTTTGGCCCTGAGAAAGTCCCAGTA AGAAGTT

Energy 32: -19.155000 (kcal/mol): Start=6589 S1=5 S2=6 L1=2 L2=14 L3=0 End=6626

~~GTGCTTGCGGTCAG AA~~

~~TCGGT CGTTAG~~

~~GTTTCCTCCCAACGCCAATTAGCCG GTGATCCC~~

~~Energy 33: -18.950000 (kcal/mol): Start=5855 S1=7 S2=6 L1=1 L2=49 L3=3 End=5930~~

~~ACCTGGAGCTTAGTGATAATATCTCAAATATTCCAAGTTCAAGCCGGTT A~~

~~CTTTACT ACT AACTTC~~

~~TGCCTTTTCCTTTGCTCACAGAGATGA TTGGAGAT~~

Energy 34: -18.930000 (kcal/mol): Start=3 S1=7 S2=5 L1=5 L2=46 L3=1 End=77

ATGTCTTGGCCTCTGTTTGCTCTAGCTCTACGCTTAAGATTCAAGA AGCCG

GTAGGCC C GTCGT

CCTGTCCGG TGGCGAA

Energy 35: -18.930000 (kcal/mol): Start=7826 S1=7 S2=5 L1=5 L2=46 L3=1 End=7900

ATGTCTTGGCCTCTGTTTGCTCTAGCTCTACGCTTAAGATTCAAGA AGCCG

GTAGGCC C GTCGT

ATATATTTAAAAGGGTGACCTGTCCGG TGGCGAA

```
Energy 36: -18.740000 (kcal/mol): Start=3095 S1=5 S2=7 L1=2 L2=44 L3=1 End=3164
                    GTCACTGCCCAAATGTTAGCCCAAGGCTACAGCCCCGGAAAAGG       AG
                    ATACA  G  TGATCCT
TTCTCAAATGAAAATTATGATGTGT     ATTAGGAAA

Energy 37: -18.720000 (kcal/mol): Start=1739 S1=6 S2=6 L1=8 L2=27 L3=2 End=1797
                    CTCAAATTCAAGCGGCTGCTACAAAAG       CAATATGA
                    GTTTGT  TA  GGGCCT
ACTATTCTAGCACTGATGCACAAATG      CCTGGAGA

Energy 38: -18.600000 (kcal/mol): Start=1286 S1=5 S2=5 L1=6 L2=49 L3=1 End=1360
                    TTTCTCGCACACCTCACTGGCCGGGGCAACATATCCCTAAAGGAAAATG       AGAAAC
                    TCGGG  G  GACGT
CAGGAAATGAAACTGTCACTAGTCC     CTGCGCC

Energy 39: -18.540000 (kcal/mol): Start=624 S1=5 S2=6 L1=3 L2=3 L3=2 End=651
                    AGG       CCT
                    GAACG  CC  TTTGGT
TTTTGATTTTGTAAAGGATACTTGT      GAACCATA

Energy 40: -18.480000 (kcal/mol): Start=3088 S1=6 S2=7 L1=8 L2=42 L3=2 End=3163
                    CACTGCCCAAATGTTAGCCCAAGGCTACAGCCCCGGAAAACG       ATGTGTAG
                    TGATAC  AG  TGATCCT
ATCTCCTTTCTCAAATGAAAATTATG      ATTAGGAAA

Energy 41: -18.440000 (kcal/mol): Start=4613 S1=5 S2=6 L1=7 L2=28 L3=0 End=4669
                    CATATACTCTTGCTGATACTACCATCAA       CTTCCAC
                    GCCGG    TAAGGT
GCTTTATTAGTTTTTACTGATGGCT    ATTTCAAA

Energy 42: -18.430000 (kcal/mol): Start=1656 S1=5 S2=5 L1=8 L2=6 L3=1 End=1689
                    GTAATG       AAAAGAAA
                    GCCGA  A  CAACT
TTTTGAAAATTGTAGAGAAACGGCT     GTTGGGA
```

```
Energy 43: -18.410000 (kcal/mol): Start=2150 S1=6 S2=6 L1=5 L2=50 L3=3 End=2228
                    TTTTGCAAAAAATTGTCATGAACATATACATAACAATTCTGAAACAAAGG       AAATG
                    ACAGGA  AAG  GAGGGC
ATAAAGAAAAAGGAGGGTGTTGTTTT       CTCCTGGA

Energy 44: -18.285000 (kcal/mol): Start=387 S1=5 S2=5 L1=8 L2=44 L3=0 End=458
                    CGGCCCGGATTAAAAGAGAAACGAAAGTAAACTTTCTTCGGCCG       GAAGACGA
                    CGTTT    GGCGC
GTTGGATACGAGGGAATTTCGTGAG    CCGCGGG

Energy 45: -18.270000 (kcal/mol): Start=7268 S1=7 S2=5 L1=1 L2=19 L3=0 End=7311
                    AGCTTTACAGGAAAAATGC       G
                    TTCGTCT    ACGGA
GATTAGATCTGCTGACAGCAGAGCAGG    TGTTTCT

Energy 46: -18.215000 (kcal/mol): Start=7074 S1=5 S2=5 L1=9 L2=5 L3=0 End=7107
                    CCGGG        ATAACTACT
                    ATCTA    TGACG
TATTCCCCTAGTCATAGGATTAGGT    ACTGCTG

Energy 47: -18.080000 (kcal/mol): Start=7052 S1=5 S2=7 L1=1 L2=40 L3=2 End=7116
                    ATTAGGTATAACTACTGCAGTATCTACCGGGACTGCTGGT       T
                    GGATA  CT  GATCCCC
GACCCAAAAGAGCAATCCAGTTTAT      CTGGGGGTT

Energy 48: -18.030000 (kcal/mol): Start=6358 S1=5 S2=5 L1=5 L2=15 L3=0 End=6397
                    ACATGTCTGATGGAG       GCCAA
                    CTTGT    CTCCC
AAGGGCCAAGTGGTTTGCTGGAATA    GAGGGCC

Energy 49: -18.020000 (kcal/mol): Start=1813 S1=8 S2=5 L1=3 L2=37 L3=2 End=1878
                    CAGGAGTCAAACAAGGACCCGATGAGCCATTTGCAGA       ACC
                    ATTCCCTT  CG  AGGAC
GCCTGGAGAAAACTTCCCGTTAAGGGAG      TTTTGTA
```

Energy 50: -18.000000 (kcal/mol): Start=4357 S1=5 S2=5 L1=4 L2=49 L3=2 End=4429

```
                    AAAAATACTTTGGAATTGAACCCTCCGTAATCATACAGCCATACTCTAA       GGAA
                    ATGAT  AC  CAGAG
CGCTATAGCAGATTTAATCATACTA      GTCTCAA
```

Time taken to run: 4716 seconds 350 milliseconds

A. The HTLV group

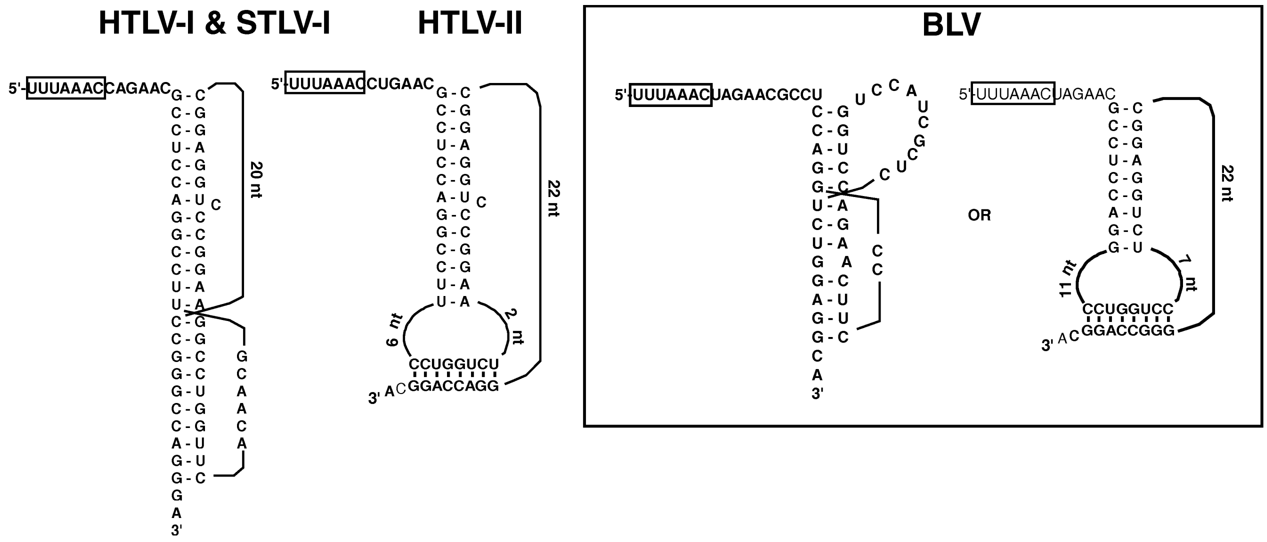

B. The SRV group

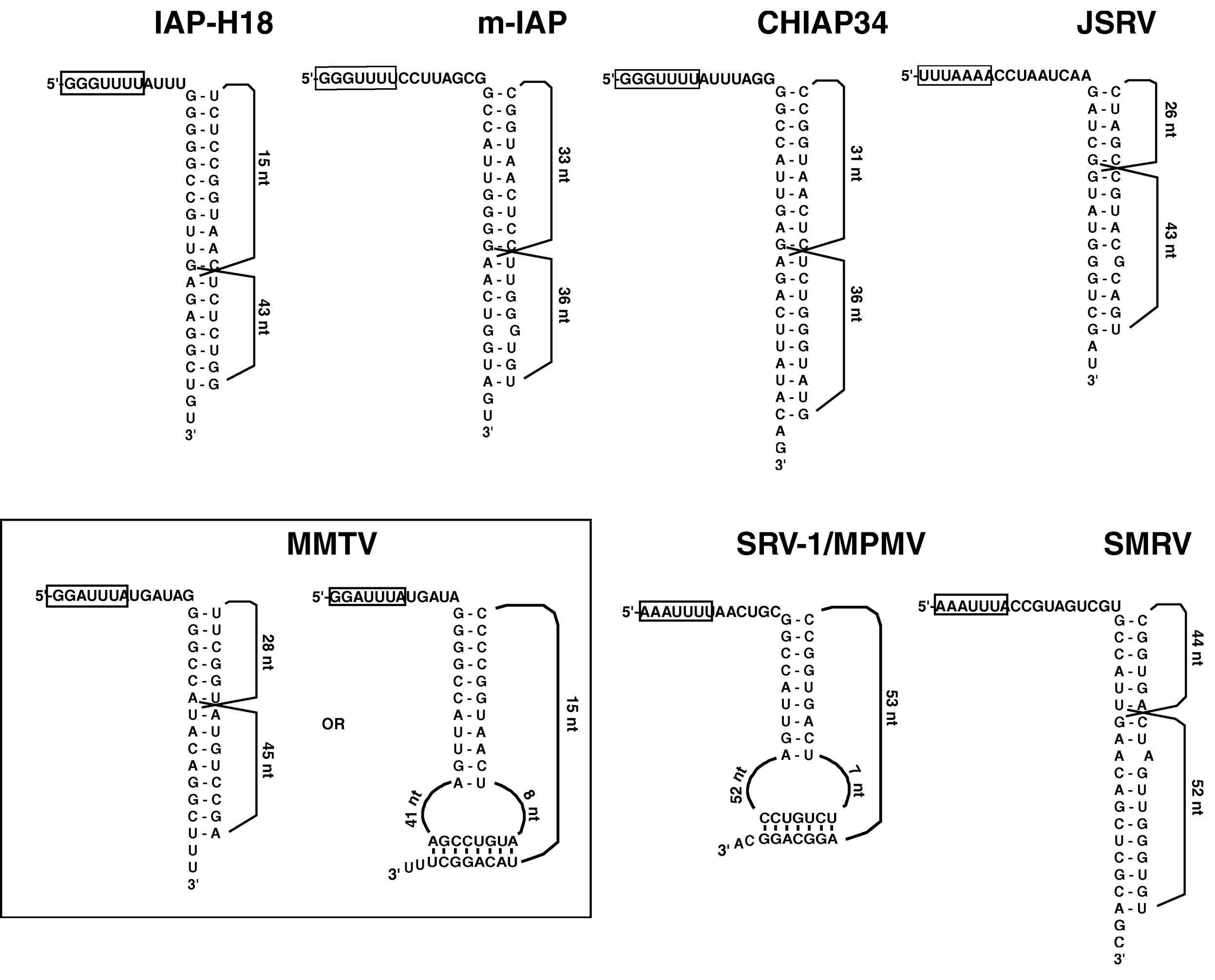

Supplement: Supplementary file 1 — In a computer-generated output (figure below), the sequence elements of the detected pseudoknot (See Figure 1 of the article for sequence elements of a pseudoknot) and a sequence of 20 nt preceding the pseudoknot are shown in three lines. The first line is for L2 and L1. The second line is for S1-3' and S2-5' sequences. The third line is for the 20 nt preceding sequence (which may contain the slippery sequence and spacer region), S1-5', and S2-3' sequences. Note that the 5'→3' orientation of the sequence in the second line runs from the right to the left, which is opposite to the sequences in the other two lines. The free energy (∆Go37) values for the two stems S1 and S2 are calculated and indicated. The output also indicates the starting/ending positions of the pseudoknot-forming sequence, the number of nucleotides in the stems/loops (S1, S2, L1, L2, and L3) of the pseudoknot. The detected pseudoknots in a viral genome are ranked by the calculated free energy values. In the sample output, some pseudoknots have their pseudoknot-forming sequences overlapped (such as the first and second ranked pseudoknots), meaning that the pseudoknots cannot exist simultaneously. The overlapped pseudoknots with a higher free energy are indicated by a line through the middle of the selected text. [file 984028.f1.pdf]
